# The MAPK MEK1/2-ERK1/2 Pathway and Its Implication in Hepatocyte Cell Cycle Control

**DOI:** 10.1155/2012/328372

**Published:** 2012-10-24

**Authors:** Jean-Philippe Guégan, Christophe Frémin, Georges Baffet

**Affiliations:** ^1^Inserm, U1085, Institut de Recherche sur la Santé l'Environnement et le Travail (IRSET), Université de Rennes 1, SFR Biosit, 35043 Rennes, France; ^2^IRSET, Université de Rennes 1, Campus de Villejean, CS 34317, 35043 Rennes, France; ^3^Institut de Recherche en Immunologie et Cancérologie, Université de Montréal, P.O. Box 6128, Montreal, QC, Canada H3C 3J7

## Abstract

Primary cultures of hepatocytes are powerful models in studying the sequence of events that are necessary for cell progression from a G0-like state to S phase. The models mimic the physiological process of hepatic regeneration after liver injury or partial hepatectomy. Many reports suggest that the mitogen-activated protein kinase (MAPK) ERK1/2 can support hepatocyte proliferation *in vitro* and *in vivo* and the MEK/ERK cascade acts as an essential element in hepatocyte responses induced by the EGF. Moreover, its disregulation has been associated with the promotion of tumor cell growth of a variety of tumors, including hepatocellular carcinoma. Whereas the strict specificity of action of ERK1 and ERK2 is still debated, the MAPKs may have specific biological functions under certain contexts and according to the differentiation status of the cells, notably hepatocytes. In this paper, we will focus on MEK1/2-ERK1/2 activations and roles in normal rodent hepatocytes *in vitro* and in vivo after partial hepatectomy and in human hepatocarcinoma cells. The possible specificity of ERK1 and ERK2 in normal and transformed hepatocyte will be discussed in regard to other differentiated and undifferentiated cellular models.

## 1. Introduction

Adult hepatocyte has a long lasting life and rarely divides in normal conditions. However, under certain situations of stress as viral infection, toxic injury, and partial hepatectomy, they can divide in reaction to the loss of liver mass. Among these different situations, the regeneration of liver after partial hepatectomy (PHT) provides an *in vivo* model to dissect the mechanisms of control of a highly differentiated normal cell growth. Indeed, surgical removal of 70% of the liver synchronized most hepatocytes and the cell cycle is characterized by a fast G0/G1 phase transition of the cell cycle after PHT, followed by a well-synchronized long G1 phase [1–3]. There is an initial step priming phase, in which the activation of IL6 and TNF alpha pathways allows hepatocytes to undergo the transition from G0 to G1 *in vivo* leading to activation of NF-kB, AP-1, and STAT3. Then, hepatocytes proliferation is regulated by different mitogens including HGF, IGF1, ligands of the EGF, and FGF receptors [4, 5]. *In vitro*, hepatocytes can also proliferate after growth factor stimulations and *in vitro* rat hepatocyte cell cycle progression highly mimicked the kinetic of cell proliferation during liver regeneration after PHT [6, 7]. In response to mitogens (i.e., EGF, HGF, PDGF, TGF alpha), hepatocytes maintained in short-term culture can undergo one or two rounds of replication (for reviews see [4, 8, 9]). This model has been extensively used by many laboratories illustrating that primary culture of hepatocytes can be a powerful model to study the precise sequences of events which are necessary for hepatocyte cell cycle progression from a G0-like state to S phase. 

There are four MAPK families categorized by sequence homology and functions: ERK1/2, p38, JNK, and ERK5. Mostly, JNK and p38 are more activated in response to cellular stress and cytokines. Numerous studies have shown that growth factor could enhance cell proliferation and survival through the activation of the MEK1/2-ERK1/2 pathway, including hepatocytes in primary culture. Indeed, the ERK1/2 are activated in response to external and internal stimuli in numerous cell types and play a central role in many signal transduction pathways. The Ras-Raf-MEK1/2-ERK1/2 pathway couples signal from the cell surface receptors to cytoplasmic substrates and transcription factors, which regulate gene expression [10–12]. Following binding of growth factors, cytokines, or extracellular matrix proteins to their receptors, activation of the cascade can occur. The pathway involves the activation of the MEK1/2, by c-Raf which in turn, activates ERK1/2. ERK1/2 can directly phosphorylate many targets (over 160) including transcription factors (e.g., Ets-1, c-Jun, c-Myc, P53) which leads to the induction of many cell cycle proteins (e.g., p21, Cyclin D1, cdk1). ERK1/2 can also phosphorylate and activate cytoplasmic substrates like the 90 KDa ribosomal S6 kinase (P90 RSK) which leads to the activation of the CREB transcription factor, apoptotic factors (e.g., caspase 9, bad, Bim), and also contribute to a mechanism of retrocontrol of the cascade by phosphorylation of the EGFr, Sos, and Raf. In addition to proliferation, the Ras-Raf-MEK1/2-ERK1/2 cascade can antagonize cell death and activate survival signals. Aberrant activation of this pathway is frequently observed in human HCC [13–16]. The MEK-ERK pathway has been implicated in the regulation of both G1/S and G2/M transitions and mitosis in somatic cells. Whereas the possible specificity of MEK1 and 2, ERK1 and 2 isoforms are still in debate, and disruption of ERK2 leads to embryonic lethality early in mouse development after the implantation stage [17]. Conversely, ERK1 Knockout mice are viable and fertile [18], arguing for possible different roles of each kinase or/and that ERK gene dosage is essential and could drive their apparent biological differences. 

## 2. Mechanisms in the Sequential Control of Cell Morphology and G1 Phase Progression Involve MEK-ERK Activations in Normal Hepatocytes

There is an agreement that during liver regeneration, JNK activation is an early event [19] while activation of ERK1/2 occurs in early and mid-late G1. P38 is present in normal liver and rapidly inactivated after PHT suggesting a permissive role in DNA replication [20]. These last ten years, our laboratory has studied the role of the MEK1/2-ERK1/2 pathway in the regulation of the cell cycle and survival of hepatocytes stimulated by the EGF. We looked at long-term survival, control of multiple cell cycles, apoptosis engagement of normal rodent hepatocytes, and rat and human hepatocarcinoma cell lines, *in vitro* and *in vivo*. The MEK1/2-ERK1/2 cascade is activated at two points of the G1 progression in mature rat hepatocytes [21]: the first one occurs in early G1 after PHT; the second one occurs in mid-late G1 phase and is associated with the induction of cyclin D1 [22], a cyclin associated to late G1 phase progression of many cells including hepatocytes [23, 24]. *In vitro*, during tissue disruption by collagenase, hepatocytes can enter into the G1 phase and undergo, depending on the culture conditions in primary culture, at least one round of division [6, 7, 25]. In the absence of growth factor, rat hepatocytes are blocked at 2/3 of G1 phase and rapidly progress through apoptosis [26, 27]. The growth factor (i.e., EGF) is a morphogen in early G1 phase by inducing controlled spreading of hepatocytes via a MEK/ERK-integrin *β*1 regulation, *in vitro* [28]. During hepatocyte spreading, Rac1 trough NADPH oxidase is part of the signalling pathway constituted by FAK-Rac1-ERK that regulates focal adhesion disassembly important for the turnover of adhesion sites that leads to cell spread [29]. The growth factor-induced nuclear translocation of ERK is an adhesion-dependent event and requires signalling from Rac1 [30]. Cell spread and migration are dynamic processes involving the focal adhesion assembly/disassembly and ERK1/2 are activated downstream of FAK while ERK1/2 can mediate its phosphorylation [31].

**Scheme 1:**
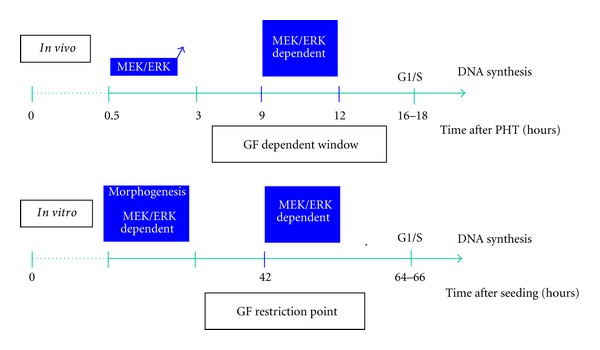
*In vivo* and *in vitro* MEK1/2-ERK1/2 dependencies during G1 phase progression.

A mitogenic effect occurs in mid-late G1 phase and allows hepatocytes to progress through a growth factor restriction point at two thirds of the G1 phase [32]. MEK signaling cascade is essential for progression to late G1 phase *in vitro* as well as *in vivo* after PHT [21]. Indeed, a growth factor-MEK dependency could be defined in mid-late G1 phase in regenerating liver between 9 and 12 h after PHT. This activation controls expression of cyclin D1 and cdk1 which are upregulated in the prereplicative phase of liver regeneration and in proliferating hepatocytes *in vitro*. Very interesting results from the Hansen's lab have demonstrated that adhesion to polymerized collagen could induce growth arrest by inhibiting the Ras/ERK pathway to cyclin D1 required in late G1 [33, 34]. Moreover, the involvement of the cell shape/motility via an ERK-MLCK-P70S6 K-dependent regulation of G1/S was specified in proliferating hepatocytes [35] and in other cell types [31, 36, 37]. All these results highlight the mechanisms by which a growth factor can temporally control morphogenic and mitogenic signals during G1 phase progression (see Scheme 1). A precise location in the cell cycle appears determinant for the regulation of ERK1/2 pathway and sequential checkpoints in early G1, mid-late G1, and G1/S transition control hepatocyte cell cycle progression, making them permissive for DNA replication.

The signaling crosstalk is an important aspect of the regulation of liver regeneration and other pathways (i.e., HGF/c-MET, IGF1/IGF-R, GH) are activated and required for efficient liver regeneration. Indeed, GH receptor KO impaired regeneration with a downregulation of ERK1/2 activation [38]. Liver regeneration and ERK pathway are also impaired in mice with liver-specific knockouts of IGF-1R or IGF binding protein 1 [39, 40]. HGF and IGF-1 strongly activated AKT and ERK1/2 *in vitro* [41]. *In vivo*, EGF and HGF have been implicated in liver regeneration, but specific deletion of EGF receptor in hepatocyte led to liver regeneration deficiencies after 2/3 PHT in mice without activation defect of ERK1/2 while p38 MAPK and NF-kB activation was reduced in regenerating mutant livers, indicating an impaired stress response after hepatectomy [42]. Indeed, p38 MAPK could play a permissive role in DNA replication during liver regeneration consistent with a role in the maintenance of hepatocyte cell cycle arrest in adult liver [20], while JNK could be involved in the G0/G1 transition [19]. Interestingly, hepatocyte deletion of c-Met which led to liver regeneration defect was associated with MEK-ERK pathway inhibition highlighting that HGF contributes dominantly to ERK1/2 activation *in vivo* [43, 44]. A persistent EGF supplementation *in vitro* only partially rescues the effect of ERK1/2 downregulation in c-MET depleted hepatocytes and restores to some extent DNA synthesis and protein levels of cdk1, Aurora A and B, and Mad2 [44].

## 3. Transient Blockade of the MEK/ERK Pathway Using Allows Multiple Cell Cycles

Different *in vitro* models have previously described that hepatocytes can undergo several cell cycles in primary cultures and long-term survival when appropriate culture conditions are provided [45–52]. Indeed, removal of EGF in long-term survival DMSO culture conditions followed by readdition of the growth factor was accompanied with an increase in DNA synthesis, and multiple round of replication could be observed by alternating addition/removal [53–55]. In coculture with liver biliary cell [56–60], EGF alone prolonged cell progression up to late G1 phase, whereas TNF*α* mediated extracellular remodeling is required for multiple division cycles [51]. Interestingly, TNF*α* promoted an extracellular matrix degradation required for initiating a new hepatocyte division wave. Furthermore, a network of ECM or polymerized collagen type I gel induces a highly differentiated but growth-arrested phenotype in primary cultures, whereas a film of collagen promotes cell cycle progression and dedifferentiation [34, 61, 62]. Hepatocytes dedifferentiation is reversible in consequence of a specific network triggered by the extracellular matrix, an active process driven by FAK-mediated AKT and ERK1/2 signaling [63]. As well, in hepatocellular carcinoma cells, increasing matrix stiffness promotes proliferation whereas soft environment induces cellular dormancy [64].

**Scheme 2:**
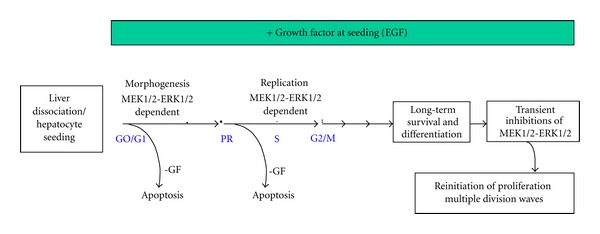
Long-term survival/differentiation in EGF stimulated cells and sequential MEK/ERK inhibitions allowing multiple divisions waves. Hepatocytes are cultured in presence or absence (-GF) of EGF and after one week of culture, transient inhibitions of MEK/ERK by U0126 allow EGF-cultured hepatocytes to re-enter in new cell cycles.

All these experiments and others indicated that adult hepatocytes could undergo long-term survival and multiple cell divisions. In this context, our group have demonstrated that rat hepatocytes seeded in the presence of EGF (in the absence of FCS) increased cell spreading [28] and greatly enhanced cell survival [65]. However, only one peak of BrdU incorporation was obtained in EGF-seeded cultured whereas nearly 100% of the hepatocytes accomplished a complete cell cycle. Time-lapse cinematography showed that both mononuclear and binuclear hepatocytes underwent mitosis [66]. Some reports have suggested that sustained activation of ERK inhibits hepatocyte DNA replication and that transient activations of this pathway could stimulate DNA synthesis [67, 68]. We therefore hypothesized that maintained MEK1/2-ERK1/2 stimulation of hepatocytes by EGF could lead to a sustained activation of ERK responsible for the negative control of the progression in a second cell cycle. Indeed, when the MEK/ERK pathway is transiently inhibited with the MEK inhibitor U0126 about 60% of hepatocytes did replicate their DNA showing that primary hepatocytes are able to perform 2 cell cycles when a break of the MEK/ERK signalling pathway activity is done [66]. In addition, cyclin D1, E, A2, cdk1, P21, and P27 were downregulated in MEK-inhibited cells and induced after the U0126 removal. A third peak of DNA synthesis in EGF-seeded hepatocytes by performing another 2 days-break of MEK1/2-ERK1/2 activity could be obtained demonstrating that EGF-seeded hepatocytes were able to perform multiple division waves after sequential MEK1/2-ERK1/2 pathway inhibitions (see Scheme 2). 

## 4. Early Sustained EGF Stimulation and MEK Inhibition Maintain Hepatocytes in a Long-Term Survival and Differentiated States

In hepatocytes, in addition to its proliferating properties, EGF could induce survival. *In vitro*, in the absence of serum and growth factor stimulation, hepatocytes in primary culture adhered to the plastic support but underwent spreading with a very low efficiency and die as observed by rapid caspase3/7 activations evaluated using a DEVD-AMC assay [65]. At the opposite, hepatocytes seeded with EGF alone and cultured with the growth factor all along the culture time present a high level of differentiation. Cell survival can be maintained at least 15 to 20 days in this culture condition. Albumin expression reached a level closed to half of freshly isolated cells, and CYP450s can be induced by 3MC or PB showing that the detoxification machinery is still fully operative. In these cells, ERK localization could be determinant for the cell phenotype since Rosseland et al. showed that the cytoplasmic retention of transient peroxide-activated ERK provides survival in primary cultures of rat hepatocytes [69]. Indeed, MEK1 and MEK2 could regulate distinct functions by sorting ERK2 to different intracellular compartments in response to growth factor and ERK2 intracellular localization could determine whether growth factors mediate hepatocyte proliferation or survival in an adhesion-dependent manner [70–72].

Surprisingly, an improvement of the survival of hepatocytes continuously treated with the MEK inhibitor U0126 can be obtained [65]. Indeed, a permanent treatment with U0126 keeps hepatocytes for more than 2 weeks in survival. All the genes of detoxification analyzed (Cyp 1A1, 1A2, 2B2, 3A23, and GSTa2) as well as the aldolase B gene are induced all along the period of treatment. U0126 removal from the culture medium is accompanied with a fast decrease of the expression of these markers related to the reentry of the cells in a new cycle. 

In summary, early and sustained EGF stimulation, in the absence of serum, could be a good compromise between “classical monolayers” with limited survival/differentiation, and long-term sophisticated and labor intensive cultures. This model emphasizes that early EGF stimulation of hepatocytes in the absence of FCS and transient or sustained inhibition of the MEK/ERK pathway represent serum-free models (Scheme 2) that will be very helpful for pharmacological studies on drug metabolism and toxicity.

## 5. Specificity of the MAPK ERK1 and ERK2 Signaling: ERK2 Controls S Phase Entry whereas ERK1 Regulates Survival in ****Hepatocyte

The strict specificity of action of the MAPKs is still debated and today, no one can affirm with certitude the full redundancy of ERK1 and ERK2 or at the contrary the specificity of action of each protein. On one hand, the simple observation of the phenotypes of knockout animals for ERK1 and ERK2 fuels the idea that each ERK isoform could regulate specific and non overlapping functions. Invalidating ERK1 has no strong and lethal impact on animals: mice are viable, fertile, and of normal size [18]. Actually, only a few defects affecting different cell lineages have been related. Thereby, ERK1 was associated with maturation of thymocytes [18], development of adipose tissue [73], or osteoclast formation and differentiation [74, 75]. ERK2 knockout is much more severe as embryos die very early during development [17, 76], because of major defects in the establishment of extraembryonic tissues [17, 76, 77]. To counteract this embryonic lethality and ascertain the roles of ERK2 in embryo or adult tissues, conditional expressions have been used allowing the invalidation of ERK2 in specific sites. Invalidation of *Erk2* gene in the neural crest induces craniofacial and cardiac defects [78]. ERK2 also regulates multiple stages of T-cells development [79, 80]. Invalidation of ERK2 in the central neural system (CNS) leads to anomalies in multiple aspects of social behaviors, decreased anxiety-related attitude, and impaired long-term memory [81]. ERK2 also protects the myocardium from ischemia-reperfusion injury *in vivo* as *Erk*2^+/−^ gene-targeted mice showed enhanced infarction areas [82]. Based on the strict observation of these phenotypes, one could easily conclude that ERK1 and ERK2 regulate specific functions.

Indeed, studies performed on animal and which attributed to ERK1 or ERK2 unique functions did not really took into account the expression level of each isoform in the tissues or cell types analyzed. In other words, the lethality of ERK2 embryos could reflect a specific role of the isoform in the establishment of extraembryonic tissues or could be due to a higher expression of the ERK2 isoform (compared to ERK1) in these tissues. We must be careful when drawing some conclusions about specific roles for ERK1 and ERK2. Besides, on a purely biochemical point of view, it has been difficult to associate ERK1 or ERK2 to cellular specific functions. They share a 84% homology at the protein level, seem to be activated in response to similar stimuli and to date no specific substrate for each kinase has been characterized. Actually, only a few papers have reported biochemical differences between both proteins. Thus, preferential activation of ERK1 versus ERK2 was reported in NFB4 cells after LPA stimulation [83]. At the contrary, activation of ERK2 rather than ERK1 occurs during thrombin-stimulated platelet activation [84]. Another biochemical difference concerns the identification of a specific scaffolding protein of ERK1 called MP1 (MEK partner 1). This protein interacts exclusively with MEK1 and ERK1 at the surface of late endosome [85, 86]. Finally, despite the fact that both kinases are simultaneously expressed in all cell types and tissues analyzed, the ERK1 : ERK2 ratio is quite variable. One of the best examples that illustrates this is the quite heterogenous expression profile of ERK1 and ERK2 mRNA in brain [87]. These are essentially the more pronounced biochemical differences that have been reported until today and finally ERK1 and ERK2 appear as tightly close enzymes. 

Interestingly, ERK is highly activated in ectoplacental cone and extraembryonic ectoderm, which both give rise to these extraembryonic tissues [88]. Even if the elevated ERK activity in these tissues has not been attributed to ERK1 or ERK2, it is likely to be mainly carried by ERK2 isoform, which would explain the phenotype of ERK2 knockout. It is indeed assumed that ERK2 is more expressed than ERK1 in nearly all tissues examined so far and, as a consequence invalidation of ERK2, is supposed to have a stronger impact on the global ERK activity compared to ERK1 knockout. Actually, the only one way to compare the expression levels of ERK1 and ERK2 in cells is the use of a phosphospecific antibody, which recognizes the phosphorylation of activation loop residues Thr202/Tyr204 and Thr185/Tyr187 of ERK1 and ERK2, respectively. This motif is recognized with the same affinity by the antibody. In a recent report, Lefloch et al. have established a clear correlation between the expression ratio of ERK1 and ERK2 and their activation ratio [89]. In this work, the authors have demonstrated that ERK1 and ERK2 are fully redundant kinases regarding the regulation of cell proliferation in NIH3T3 cells. A similar observation was done on embryonic fibroblast genetically deficient for ERK1 and/or ERK2 [90]. In this study, a strong correlation was drawn between the quantity of ERK proteins inside the cell and the intensity of proliferation. 

In addition to these works based on the use of genetically deficient animals and riding the wave following RNAi discovery, a sustained number of studies have emerged in the literature in order to decipher the roles of ERK1 and ERK2. In skeletal myoblasts, proliferation requires one of the two isoforms, whatever it is, but terminal differentiation is strictly dependent on ERK2 [91]. According to Liu et al., if ERK1 and ERK2 silencing would both affect cell proliferation, each kinase would be involved at different phases of the cell cycle: ERK1 would regulate G2/M transition while ERK2 could be essential in G1 phase [92]. A recent study done by John Blenis' group has shown that the ERK2 isoform induces epithelial-to-mesenchymal transformation when overexpressed [93]. Interestingly, ERK1 is not able to reach the same effect despite high expression levels. 

What about the roles of ERK1 and ERK2 in the physiology of normal hepatocytes? Interestingly, contrary to other cell types in which ERK2 is much more expressed compared to ERK1, normal hepatocytes harbor closely similar levels of ERK1 and ERK2, according to P-ERK1 : P-ERK2 ratio. We have shown that ERK1^−/−^ hepatocytes proliferate with similar kinetics as wild-type hepatocytes after *in vivo* PHT and *in vitro* [94]. At the contrary, silencing ERK2 has a strong impact on cell proliferation [66, 94]. These results are in accordance with several papers listed above that place ERK2 as a positive regulator of cell proliferation. We went further in the analysis of putative roles of ERK1 and ERK2 in hepatic processes and established a link between ERK1 and the cell survival of hepatocytes. Indeed, silencing ERK1 using RNAi decreases susceptibility to apoptosis as it is observed in ERK1-deficient hepatocytes. This is in accordance with the report from Bourcier et al. which has shown that ERK1^−/−^ keratinocytes are resistant to apoptosis induced by different agents or stress [95]. Interestingly inhibiting ERK2 did not reach this effect. But this is probably not so simple and associating one isoform to the regulation of one specific cellular function could be a dangerous shortcut. As an example, in ovarian cells, the silencing of ERK1 triggers the opposite phenotype to that observed in hepatocytes since cells become more sensitive to apoptosis [96]. How the inhibition of the same protein could have radically opposite effects? The localization of the isoforms inside the cell could be a response element. In that sense, MEK1 and MEK2 were proposed to regulate distinct cellular functions in hepatocytes by localizing ERK2 to different regions of the cell [97]. Following activation by MEK1, ERK2 translocates to the nucleus where it would trigger a proliferative response. At the opposite, when activated by MEK2, ERK2 retains a cytoplasmic localization to mediate survival. This could explain why one isoform would regulate distinct functions according to the cell type.

Finally and in order to reconcile all these data, one can easily imagine that ERK1 and ERK2 regulate overlapping fundamental functions with regard to the most fundamental processes such as proliferation in nonspecialized cells. Results obtained on MEFs cells and which explicit a dose-response effect of ERK on the intensity of cell proliferation argue in that sense [98]. Interestingly, all the major differences that were observed between ERK isoforms have been made on more differentiated cells, and notably hepatocytes. This could suggest a specialization of ERK1 and ERK2 in the regulation of unique biological functions in differentiated cells. ERK1 and ERK2 also share dozen of substrates so one could speculate that potential differences in substrate affinity could explain the predominant role of one isoform in a function due to the presence of a particular set of partners, in other words in a particular cellular context.

## 6. Roles of the MEK1/2-ERK1/2 Pathway in ****Hepatocellular Carcinoma

The MEK/ERK signaling pathway plays a central role in the regulation of various physiological processes such as proliferation, survival or cell motility. Thus, its disregulation has often been associated with the promotion or development of tumor cell growth. Indeed, the chemical inhibition of MEK1/2 kinase activities blocks *in vitro* as well as *in vivo* proliferation of a variety of tumor models, including hepatocellular carcinoma (HCC) [99–103]. Moreover, active mutant forms of Raf or MEK have been shown to transform different cell types [90, 104–106]. The best illustration of MAPK pathway importance in oncogenesis lies in the observation of an ERK1/2 overactivation in 50 out of 138 human tumor cell lines [107]. Indeed, an increased expression and activation of the MEK1/2, and ERK1/2 kinases has been reported in human and mice primary liver tumors [108–111]. Active forms of the MEK/ERK pathway components including pRAF1, pMEK1/2 and pERK1/2 were also associated with poor prognosis in patients with HCC [112, 113]. However, in HCC, the overactivation of the MEK/ERK pathway did not result (or rarely) from an activating mutation of an upstream protein, namely, the GTPase Ras or the Raf protein kinase. Generally, the protein Ras is found mutated in about 30% of all human cancers with a high prevalence in the pancreas (90%) and colon (50%) adenocarcinomas [114]. The BRAF V600E mutation is on the other hand found in about 20% of tumors and especially in melanoma (~ 50%) [115]. In HCC, Ras and BRAF mutations are rare in humans or could be related to some etiologic factors or genetic backgrounds [116–119]. Overactivation of the MEK/ERK pathway is rather a consequence of a disinhibition, an upregulation of upstream activators, or an oncogenic stimulation.

For example, Calvisi et al. have shown from 80 surgical resections of HCC that 100% of tested malignant tissues had a constitutive activation of Ras and this was linked to the loss or reduction of the Ras inhibitory proteins NORE1A and RASSF1A [120]. Different inhibitors of the MEK/ERK pathway like RKIP, Sprouty-2, Spred-1, or Spred-2 are also frequently downregulated in human tissues of HCC. These decreases, or losses of activity, are supposed to have an important impact on HCC development and progression since ectopic expression of these different inhibitors is sufficient to inhibit the MAPK pathway but, most of all, to suppress tumor cell proliferation both *in vitro* and *in vivo* [121–124]. The downregulation of the previously cited inhibitors would provide an increased activation of the kinases ERK1/2. Moreover, in poor prognosis HCC, ERK1/2 activity should also be unrestrained given the weakened expression of the MAPK phosphatase DUSP-1 found in those patients [125].

In addition, the ERK1/2 kinases could also undergo a more intense stimulation through the overexpression of various components of the MEK/ERK pathway. For instance, the protein c-Raf could be an important source of ERK overactivation. Indeed, Hwang and colleagues have shown that the c-Raf kinase is upregulated in almost all cirrhosis and tumor tissues analyzed. A significantly higher level of expression of c-Raf was also reported in hepatocellular carcinomas when compared to cirrhosis [126]. Numerous tyrosine kinase receptors and their ligands also accuse an increased expression in HCC [127]. The signaling via these receptors will thus activate the MAPK pathway cascade and lead to a sustained activation of ERK1/2. For example, the EGF receptor is overexpressed in about 40% to 70% of HCC [128–130]. Importantly, the transforming growth factor-*α* which is one of the EGFR ligands presents in parallel an increased expression in cirrhosis and early HCC. Since the gene encoding for TGF-*α* is also a target of the Ras pathway, an autoamplification loop could be established ensuring, by the way, a persistent activation of the MEK/ERK pathway [131–133]. Hepatocellular carcinoma is also one of the most vascularized solid tumors links to a strong angiogenesis. Therefore, it is not surprising to find an upregulation of various proangiogenic factors such as VEGF, its receptor VEGFR, or the PDGF. All of these proteins will provide activator signals for the MEK/ERK pathway [134–136]. In HCC, a significant deregulation of the IGF signaling has also been reported, and notably a significant increase in IGF2 bioavailability. This was mediated by the upregulation of IGF2 via epigenetic mechanisms and by the downregulation of the IGF2R receptor, which normally lead to the lysosomal degradation of IGFs [79, 137, 138]. In addition, an overexpression of c-MET is observed in approximately 50% of HCC and this was associated with poor prognosis [139].

Finally, ERK1/2 overactivation in HCC could be due to Hepatitis-B virus (HBV) or C virus (HCV) infections, the two major etiologic factors of primary liver cancers. Indeed HCV carrier patients have higher rates of pERK1/2 than other HCC patients [113]. Thus, the core protein and the envelope protein E2 of the HCV and the HBX protein and preS2-activator large surface protein of HBV have been shown to directly activate the MEK/ERK pathway but by different mechanisms [140–144]. For instance, the HBX protein could upregulate the EGFR and interacts with the protein PIN1 to facilitate the dephosphorylation of c-Raf while activation by the preS2-activator large surface protein used PKC-dependent mechanisms [142, 143, 145, 146]. The envelope protein E2 of HCV stimulates the MEK/ERK pathway by binding to the CD81 receptor or to the low density lipoprotein receptor (LDLR). Anyway, MEK/ERK activation by viruses is postulated to promote hepatocarcinogenesis by facilitating the proliferation and survival of infected cells [147].

The MEK/ERK overactivation in hepatocarcinoma cells will promote various cellular processes. First, the proliferation of neoplastic cells would be obviously improved. Indeed, MEK1/2 inhibition, conducted by the use of different chemical inhibitors, could abolish the *in vitro* proliferation of numerous human and rat hepatocarcinoma cell lines. Growth of xenograft tumors in mice is also severely impaired in a context of MEK/ERK inhibition [94, 99, 104, 111, 148–152]. Using RNAi, we have specified the molecular mechanism involved in tumor hepatocyte proliferation. We have shown that MEK1 deficiency suppressed both *in vitro* and *in vivo* proliferation of Huh7 cells. On the other side, MEK2 silencing did not affect the proliferation capacity of transformed cells [151]. Similar to normal hepatocytes, tumor growth is also supported by the kinase ERK2 but not by ERK1. Indeed, ERK2 targeting by stable chemically modified siRNA altered the *in vitro* proliferation as well as the *in vivo* growth of the highly tumorigenic F1 cells. We have also demonstrated that ERK2 was primordial for the *in vivo* proliferation of the Huh-7 cell line [99, 151]. Interestingly, it is noteworthy that hepatoma cells exhibit a higher expression of ERK2 than ERK1 while normal hepatocytes have a more balanced ERK1 : ERK2 ratio. This could reflect the difference of functions carried by these kinases in the particular context of liver. Indeed, we showed that ERK2 favored the proliferation of normal and transformed hepatocytes as opposed to ERK1 which could promote a death signal [66] (Guegan, personal data). Given the permanent and sustained MEK/ERK activation in HCC, one could speculate that the newly transformed hepatocyte should thus prime ERK2 functions while diminishing ERK1 prodeath activity.

Moreover and besides its role in cell proliferation, we have shown that ERK2 but not ERK1 was involved in hepatoma cell motility and invasiveness by an uPAR and P70S6 K dependent mechanism. RNAi-mediated inhibition of ERK2 or P70S6 K led to strongly reduced cell motility [153]. However, this is not the unique mechanism by which the MAPK pathway regulates HCC invasion. Indeed Honma et al. have shown that an active mutant form of MEK1 could suppress the E-cadherin mediated homotypic adhesion and thus potentiate cell migration [154]. ERK1/2 activity was also involved in the migration of three metastatic HCC cell lines but in a PKC-*β* dependent mechanism. Interestingly, ERK activation status was shown to increase following the metastatic potential of the cell lines analyzed [155]. Hence, the MEK/ERK overactivation found in tumor cell could support the HCC progression and metastasis. 

Finally, this overactivation could also promote the survival of transformed hepatocytes. Indeed it has been shown that treatment of HepG2 or Hep3B cells by MEK1/2 inhibitors led to an apoptosis engagement [111]. Inhibition of MEK1/2 could also sensitize hepatoma cells to the death induced by ER-stress [156]. Moreover, active form of MEK1 prevented serum deprivation-induced death of hepatocarcinoma cells [157] and in HepG2, MEK/ERK activity has been reported to contribute to cisplatin induced death [158]. The MEK/ERK pathway has also been shown to protect transformed hepatocytes from TGF-*β*-induced apoptosis, a natural inducer of apoptosis in hepatocytes, produced in the liver by hepatic stellate cells [159]. The escape from TGF-*β* suppressive effects is an important step in hepatocarcinogenesis. Liver tumor bearing late TGF-*β* gene signature is indeed more aggressive than those expressing early gene signature [160]. Hence the MEK/ERK overactivation might play an important role in the initiation or development of HCC. The prosurvival effects of the MEK/ERK pathway in tumor cells have been shown to pass through the upregulation or stimulation of different antiapoptotic factors such as Bcl-2, Bim, or Bad (for review see [161]). For instance, it has been shown in hepatocarcinoma cells that ERK1/2 could phosphorylate the antiapoptotic factor Mcl-1 on thr163 in order to stabilize it and to thus enhance its prosurvival function [162]. 

The critical involvement of the MEK/ERK pathway in HCC tumorigenesis strongly suggests that the kinases MEK1/2 or ERK1/2 could be promising therapeutic targets. Sorafenib advent in therapy has also clearly demonstrated the potential of targeting signaling pathways in HCC. Given the predominant role of ERK2 in transformed hepatocyte proliferation, survival, and motility and given the prodeath role of ERK1, it could be preferential to specifically target ERK2 without affecting ERK1 activity. By this way, this might have different effects compared to a nonspecific chemical inhibition of both kinases, what could ultimately improve therapeutic benefits.

## Abbreviations

ECM: Extracellular matrix
ERK: Extracellular signal-regulated kinase
JNK: c-Jun N-terminal kinase
PHT: Partial hepatectomy
HCC: Hepatocellular carcinoma
MAPK: Mitogen-activated protein kinase
MEK: MAPK/ERK kinase
DEVD-AMC: Asp-Glu-Val-Asp-7-amino-4-methylcoumarin
FCS: Fetal calf serum
CYP: Cytochrome P450
PB: Phenobarbital
3MC: 3-methylcholanthrene
EGF: Edidermal growth factor
GH: Growth Hormone.

## References

[1] Taub R (2004). Liver regeneration: from myth to mechanism. *Nature Reviews Molecular Cell Biology*.

[2] Fausto N (2000). Liver regeneration. *Journal of Hepatology*.

[3] Michalopoulos GK, DeFrances MC (1997). Liver regeneration. *Science*.

[4] Fausto N, Campbell JS, Riehle KJ (2006). Liver regeneration. *Hepatology*.

[5] Fausto N, Campbell JS, Riehle KJ (2012). Liver regeneration. *Journal of Hepatology*.

[6] Thompson NL, Mead JE, Braun L (1986). Sequential protooncogene expression during rat liver regeneration. *Cancer Research*.

[7] Loyer P, Cariou S, Glaise D, Bilodeau M, Baffet G, Guguen-Guillouzo C (1996). Growth factor dependence of progression through G1 and S phases of adult rat hepatocytes in vitro: evidence of a mitogen restriction point in mid-late G1. *Journal of Biological Chemistry*.

[8] Diehl AM, Rai RM (1996). Regulation of signal transduction during liver regeneration. *The FASEB Journal*.

[9] Michalopoulos GK, DeFrances MC (1997). Liver regeneration. *Science*.

[10] Marshall MS (1995). Ras target proteins in eukaryotic cells. *The FASEB Journal*.

[11] McCubrey JA, Steelman LS, Chappell WH (2007). Roles of the Raf/MEK/ERK pathway in cell growth, malignant transformation and drug resistance. *Biochimica et Biophysica Acta*.

[12] Meloche S, Pouysségur J (2007). The ERK1/2 mitogen-activated protein kinase pathway as a master regulator of the G1- to S-phase transition. *Oncogene*.

[13] Schmidt CM, McKillop IH, Cahill PA, Sitzmann JV (1997). Increased MAPK expression and activity in primary human hepatocellular carcinoma. *Biochemical and Biophysical Research Communications*.

[14] Schmitz KJ, Wohlschlaeger J, Lang H (2008). Activation of the ERK and AKT signalling pathway predicts poor prognosis in hepatocellular carcinoma and ERK activation in cancer tissue is associated with hepatitis C virus infection. *Journal of Hepatology*.

[15] Huynh H, Tuyen Nguyen TT, Pierce Chow KH, Tan PH, Soo KC, Tran E (2003). Over-expression of the mitogen-activated protein kinase (MAPK) kinase (MEK)-MAPK in hepatocellular carcinoma: its role in tumor progression and apoptosis. *BMC Gastroenterology*.

[16] Ito Y, Sasaki Y, Horimoto M (1998). Activation of mitogen-activated protein kinases/extracellular signal-regulated kinases in human hepatocellular carcinoma. *Hepatology*.

[17] Saba-El-Leil MK, Vella FDJ, Vernay B (2003). An essential function of the mitogen-activated protein kinase Erk2 in mouse trophoblast development. *EMBO Reports*.

[18] Pagès G, Guérin S, Grall D (1999). Defective thymocyte maturation in p44 MAP kinase (Erk 1) knockout mice. *Science*.

[19] Schwabe RF, Bradham CA, Uehara T (2003). c-Jun-N-terminal kinase drives cyclin D1 expression and proliferation during liver regeneration. *Hepatology*.

[20] Campbell JS, Argast GM, Yuen SY, Hayes B, Fausto N (2011). Inactivation of p38 MAPK during liver regeneration. *International Journal of Biochemistry and Cell Biology*.

[21] Talarmin H, Rescan C, Cariou S (1999). The mitogen-activated protein kinase kinase/extracellular signal-regulated kinase cascade activation is a key signalling pathway involved in the regulation of G_1_ phase progression in proliferating hepatocytes. *Molecular and Cellular Biology*.

[22] Loyer P, Glaise D, Cariou S, Baffet G, Meijer L, Guguen-Guillouzo C (1994). Expression and activation of cdks (1 and 2) and cyclins in the cell cycle progression during liver regeneration. *Journal of Biological Chemistry*.

[23] Albrecht JH, Hu MY, Cerra FB (1995). Distinct patterns of cyclin D1 regulation in models of liver regeneration and human liver. *Biochemical and Biophysical Research Communications*.

[24] Koch KS, Lu XP, Leffert HL (1994). Primary rat hepatocytes express cyclin D1 messenger RNA during their growth cycle and during mitogenic transitions induced by transforming growth factor-alpha. *Biochemical and Biophysical Research Communications*.

[25] Etienne PL, Baffet G, Desvergne B, Boisnard-Rissel M, Glaise D, Guguen-Guillouzo C (1988). Transient expression of c-fos and constant expression of c-myc in freshly isolated and cultured normal adult rat hepatocytes. *Oncogene Research*.

[26] Gilot D, Serandour AL, Ilyin GP (2005). A role for caspase-8 and c-FLIPL in proliferation and cell-cycle progression of primary hepatocytes. *Carcinogenesis*.

[27] Coutant A, Rescan C, Gilot D, Loyer P, Guguen-Guillouzo C, Baffet G (2002). PI3K-FRAP/mTOR pathway is critical for hepatocyte proliferation whereas MEK/ERK supports both proliferation and survival. *Hepatology*.

[28] Rescan C, Coutant A, Talarmin H (2001). Mechanism in the sequential control of cell morphology and S phase entry by epidermal growth factor involves distinct MEK/ERK activations. *Molecular Biology of the Cell*.

[29] Flinder LI, Timofeeva OA, Rosseland CM, Wierød L, Huitfeldt HS, Skarpen E (2011). EGF-induced ERK-activation downstream of FAK requires rac1-NADPH oxidase. *Journal of Cellular Physiology*.

[30] Hirsch E, Barberis L, Brancaccio M (2002). Defective Rac-mediated proliferation and survival after targeted mutation of the *β*1 integrin cytodomain. *Journal of Cell Biology*.

[31] Klemke RL, Cai S, Giannini AL, Gallagher PJ, De Lanerolle P, Cheresh DA (1997). Regulation of cell motility by mitogen-activated protein kinase. *Journal of Cell Biology*.

[32] Loyer P, Cariou S, Glaise D, Bilodeau M, Baffet G, Guguen-Guillouzo C (1996). Growth factor dependence of progression through G1 and S phases of adult rat hepatocytes in vitro: evidence of a mitogen restriction point in mid-late G1. *Journal of Biological Chemistry*.

[33] Fassett JT, Tobolt D, Nelsen CJ, Albrecht JH, Hansen LK (2003). The role of collagen structure in mitogen stimulation of ERK, cyclin D1 expression, and G1-S progression in rat hepatocytes. *Journal of Biological Chemistry*.

[34] Hansen LK, Albrecht JH (1999). Regulation of the hepatocyte cell cycle by type I collagen matrix: role of cyclin D1. *Journal of Cell Science*.

[35] Bessard A, Coutant A, Rescan C (2006). An MLCK-dependent window in late G1 controls S phase entry of proliferating rodent hepatocytes via ERK-p70S6K pathway. *Hepatology*.

[36] Mansfield PJ, Shayman JA, Boxer LA (2000). Regulation of polymorphonuclear leukocyte phagocytosis by myosin light chain kinase after activation of mitogen-activated protein kinase. *Blood*.

[37] Nguyen DHD, Catling AD, Webb DJ (1999). Myosin light chain kinase functions downstream of Ras/ERK to promote migration of urokinase-type plasminogen activator-stimulated cells in an integrin-selective manner. *Journal of Cell Biology*.

[38] Zerrad-Saadi A, Lambert-Blot M, Mitchell C (2011). GH receptor plays a major role in liver regeneration through the control of EGFR and ERK1/2 activation. *Endocrinology*.

[39] Desbois-Mouthon C, Wendum D, Cadoret A (2006). Hepatocyte proliferation during liver regeneration is impaired in mice with liver-specific IGF-1R knockout. *The FASEB Journal*.

[40] Leu JI, Crissey MAS, Craig LE, Taub R (2003). Impaired hepatocyte DNA synthetic response posthepatectomy in insulin-like growth factor binding protein 1-deficient mice with defects in C/EBP*β* and mitogen-activated protein kinase/extracellular signal-regulated kinase regulation. *Molecular and Cellular Biology*.

[41] Matsuo R, Ohkohchi N, Murata S (2008). Platelets strongly induce hepatocyte proliferation with IGF-1 and HGF in vitro. *Journal of Surgical Research*.

[42] Natarajan A, Wagner B, Sibilia M (2007). The EGF receptor is required for efficient liver regeneration. *Proceedings of the National Academy of Sciences of the United States of America*.

[43] Borowiak M, Garratt AN, Wüstefeld T, Strehle M, Trautwein C, Birchmeier C (2004). Met provides essential signals for liver regeneration. *Proceedings of the National Academy of Sciences of the United States of America*.

[44] Factor VM, Seo D, Ishikawa T (2010). Loss of c-Met disrupts gene expression program required for G2/M progression during liver regeneration in mice. *PLoS ONE*.

[45] Miyazaki M, Handa Y, Oda M, Yabe T, Miyano K, Sato J (1985). Long-term survival of functional hepatocytes from adult rat in the presence of phenobarbital in primary culture. *Experimental Cell Research*.

[46] Tateno C, Yoshizato K (1996). Long-term cultivation of adult rat hepatocytes that undergo multiple cell divisions and express normal parenchymal phenotypes. *American Journal of Pathology*.

[47] Wu JC, Merlino G, Cveklova K, Mosinger B, Fausto N (1994). Autonomous growth in serum-free medium and production of hepatocellular carcinomas by differentiated hepatocyte lines that overexpress transforming growth factor *α*. *Cancer Research*.

[48] Runge D, Runge DM, Jäger D (2000). Serum-free, long-term cultures of human hepatocytes: maintenance of cell morphology, transcription factors, and liver-specific functions. *Biochemical and Biophysical Research Communications*.

[49] Ullrich A, Stolz DB, Ellis EC (2009). Long term cultures of primary human hepatocytes as an alternative to drug testing in animals. *Altex*.

[50] Brown LA, Arterburn LM, Miller AP (2003). Maintenance of liver functions in rat hepatocytes cultured as spheroids in a rotating wall vessel. *In Vitro Cellular & Developmental Biology*.

[51] Sérandour AL, Loyer P, Garnier D (2005). TNF*α*-mediated extracellular matrix remodeling is required for multiple division cycles in rat hepatocytes. *Hepatology*.

[52] Guguen-Guillouzo C, Corlu A (1993). Recent progresses on long-term hepatocyte primary cultures: importance of cell microenvironments. *Cytotechnology*.

[53] Isom HC, Secott T, Georgoff I (1985). Maintenance of differentiated rat hepatocytes in primary culture. *Proceedings of the National Academy of Sciences of the United States of America*.

[54] Isom H, Georgoff I, Salditt-Georgieff M, Darnell JE (1987). Persistence of liver-specific messenger RNA in cultured hepatocytes: different regulatory events for different genes. *Journal of Cell Biology*.

[55] Cable EE, Isom HC (1997). Exposure of primary rat hepatocytes in long-term DMSO culture to selected transition metals induces hepatocyte proliferation and formation of duct-like structures. *Hepatology*.

[56] Fraslin JM, Kneip B, Vaulont S, Glaise D, Munnich A, Guguen-Guillouzo C (1985). Dependence of hepatocyte-specific gene expression on cell-cell interactions in primary culture. *EMBO Journal*.

[57] Guguen Guillouzo C, Clement B, Baffet G (1983). Maintenance and reversibility of active albumin secretion by adult rat hepatocytes co-cultured with another liver epithelial cell type. *Experimental Cell Research*.

[58] Clement B, Guguen-Guillouzo C, Campion JP (1984). Long-term co-cultures of adult human hepatocytes with rat liver epithelial cells: modulation of albumin secretion and accumulation of extracellular material. *Hepatology*.

[59] Baffet G, Clement B, Glaise D (1982). Hydrocortisone modulates the production of extracellular material and albumin in long-term cocultures of adult rat hepatocytes with other liver epithelial cells. *Biochemical and Biophysical Research Communications*.

[60] Baffet G, Loyer P, Glaise D, Corlu A, Etienne PL, Guguen-Guillouzo C (1991). Distinct effects of cell-cell communication and corticosteroids on the synthesis and distribution of cytokeratins in cultured rat hepatocytes. *Journal of Cell Science*.

[61] Albrecht JH, Hansen LK (1999). Cyclin D1 promotes mitogen-independent cell cycle progression in hepatocytes. *Cell Growth and Differentiation*.

[62] Hansen LK, Wilhelm J, Fassett JT (2005). Regulation of hepatocyte cell cycle progression and differentiation by type I collagen structure. *Current Topics in Developmental Biology*.

[63] Godoy P, Hengstler JG, Ilkavets I (2009). Extracellular matrix modulates sensitivity of hepatocytes to fibroblastoid dedifferentiation and transforming growth factor *β*-induced apoptosis. *Hepatology*.

[64] Schrader J, Gordon-Walker TT, Aucott RL (2011). Matrix stiffness modulates proliferation, chemotherapeutic response, and dormancy in hepatocellular carcinoma cells. *Hepatology*.

[65] Frémin C, Ezan F, Guegan JP (2012). The complexity of ERK1 and ERK2 MAPKs in multiple hepatocyte fate responses. *Journal of Cellular Physiology*.

[66] Frémin C, Bessard A, Ezan F (2009). Multiple division cycles and long-term survival of hepatocytes are distinctly regulated by extracellular signal-regulated kinases ERK1 and ERK2. *Hepatology*.

[67] Auer KL, Park JS, Seth P (1998). Prolonged activation of the mitogen-activated protein kinase pathway promotes DNA synthesis in primary hepatocytes from p21(Cip-WAF1)-null mice, but not in hepatocytes from p16(INK4a)-null mice. *Biochemical Journal*.

[68] Tombes RM, Auer KL, Mikkelsen R (1998). The mitogen-activated protein (MAP) kinase cascade can either stimulate or inhibit DNA synthesis in primary cultures of rat hepatocytes depending upon whether its activation is acute/phasic or chronic. *Biochemical Journal*.

[69] Rosseland CM, Wierød L, Oksvold MP (2005). Cytoplasmic retention of peroxide-activated ERK provides survival in primary cultures of rat hepatocytes. *Hepatology*.

[70] Rosseland CM, Wierød L, Oksvold MP (2005). Cytoplasmic retention of peroxide-activated ERK provides survival in primary cultures of rat hepatocytes. *Hepatology*.

[71] Skarpen E, Lindeman B, Thoresen GH (2000). Impaired nuclear accumulation and shortened phosphorylation of ERK after growth factor stimulation in cultured hepatocytes from rats exposed to 2-acetylaminofluorene. *Molecular Carcinogenesis*.

[72] Skarpen E, Flinder LI, Rosseland CM (2008). MEK1 and MEK2 regulate distinct functions by sorting ERK2 to different intracellular compartments. *The FASEB Journal*.

[73] Bost F, Aouadi M, Caron L (2005). The extracellular signal-regulated kinase isoform ERK1 is specifically required for in vitro and in vivo adipogenesis. *Diabetes*.

[74] He Y, Staser K, Rhodes SD (2011). Erk1 positively regulates osteoclast differentiation and bone resorptive activity. *PLoS ONE*.

[75] Matsushita T, Yuk YC, Kawanami A, Balmes G, Landreth GE, Murakami S (2009). Extracellular signal-regulated kinase 1 (ERK1) and ERK2 play essential roles in osteoblast differentiation and in supporting osteoclastogenesis. *Molecular and Cellular Biology*.

[76] Yao Y, Li W, Wu J (2003). Extracellular signal-regulated kinase 2 is necessary for mesoderm differentiation. *Proceedings of the National Academy of Sciences of the United States of America*.

[77] Hatano N, Mori Y, Oh-hora M (2003). Essential role for ERK2 mitogen-activated protein kinase in placental development. *Genes to Cells*.

[78] Newbern J, Zhong J, Wickramasinghe SR (2008). Mouse and human phenotypes indicate a critical conserved role for ERK2 signaling in neural crest development. *Proceedings of the National Academy of Sciences of the United States of America*.

[79] De Souza AT, Hankins GR, Washington MK, Orton TC, Jirtle RL (1995). M6P/IGF2R gene is mutated in human hepatocellular carcinomas with loss of heterozygosity. *Nature Genetics*.

[80] Fischer AM, Katayama CD, Pagès G, Pouysségur J, Hedrick SM (2005). The role of Erk1 and Erk2 in multiple stages of T cell development. *Immunity*.

[81] Satoh Y, Endo S, Nakata T (2011). ERK2 contributes to the control of social behaviors in mice. *The Journal of Neuroscience*.

[82] Lips DJ, Bueno OF, Wilkins BJ (2004). MEK1-ERK2 signaling pathway protects myocardium from ischemic injury in vivo. *Circulation*.

[83] Sarbassov DD, Jones LG, Peterson CA (1997). Extracellular signal-regulated kinase-1 and -2 respond differently to mitogenic and differentiative signaling pathways in myoblasts. *Molecular Endocrinology*.

[84] Papkoff J, Chen RH, Blenis J, Forsman J (1994). p42 Mitogen-activated protein kinase and p90 ribosomal S6 kinase are selectively phosphorylated and activated during thrombin-induced platelet activation and aggregation. *Molecular and Cellular Biology*.

[85] Teis D, Taub N, Kurzbauer R (2006). p14-MP1-MEK1 signaling regulates endosomal traffic and cellular proliferation during tissue homeostasis. *Journal of Cell Biology*.

[86] Teis D, Wunderlich W, Huber LA (2002). Localization of the MP1-MAPK scaffold complex to endosomes is mediated by p14 and required for signal transduction. *Developmental Cell*.

[87] Di Benedetto B, Hitz C, Hölter SM, Kühn R, Vogt Weisenhorn DM, Wurst W (2007). Differential mRNA distribution of components of the ERK/MAPK signalling cascade in the adult mouse brain. *Journal of Comparative Neurology*.

[88] Corson LB, Yamanaka Y, Venus Lai KM, Rossant J (2003). Spatial and temporal patterns of ERK signalling during mouse embryogenesis. *Development*.

[89] Lefloch R, Pouysségur J, Lenormand P (2008). Single and combined silencing of ERK1 and ERK2 reveals their positive contribution to growth signaling depending on their expression levels. *Molecular and Cellular Biology*.

[90] Voisin L, Julien C, Duhamel S (2008). Activation of MEK1 or MEK2 isoform is sufficient to fully transform intestinal epithelial cells and induce the formation of metastatic tumors. *BMC Cancer*.

[91] Li J, Johnson SE (2006). ERK2 is required for efficient terminal differentiation of skeletal myoblasts. *Biochemical and Biophysical Research Communications*.

[92] Liu X, Yan S, Zhou T, Terada Y, Erikson RL (2004). The MAP kinase pathway is required for entry into mitosis and cell survival. *Oncogene*.

[93] Shin S, Dimitri CA, Yoon SO, Dowdle W, Blenis J (2010). ERK2 but Not ERK1 induces epithelial-to-mesenchymal transformation via DEF motif-dependent signaling events. *Molecular Cell*.

[94] Frémin C, Ezan F, Boisselier P (2007). ERK2 but not ERK1 plays a key role in hepatocyte replication: an RNAi-mediated ERK2 knockdown approach in wild-type and ERK1 null hepatocytes. *Hepatology*.

[95] Bourcier C, Jacquel A, Hess J (2006). p44 mitogen-activated protein kinase (extracellular signal-regulated kinase 1)-dependent signaling contributes to epithelial skin carcinogenesis. *Cancer Research*.

[96] Zeng P, Wagoner HA, Pescovitz OH, Steinmetz R (2005). RNA interference (RNAi) for extracellular signal-regulated kinase 1 (ERK1) alone is sufficient to suppress cell viability in ovarian cancer cells. *Cancer Biology and Therapy*.

[97] Skarpen E, Flinder LI, Rosseland CM (2008). MEK1 and MEK2 regulate distinct functions by sorting ERK2 to different intracellular compartments. *The FASEB Journal*.

[98] Voisin L, Saba-El-Leil MK, Julien C, Frémin C, Meloche S (2010). Genetic demonstration of a redundant role of extracellular signal-regulated kinase 1 (ERK1) and ERK2 mitogen-activated protein kinases in promoting fibroblast proliferation. *Molecular and Cellular Biology*.

[99] Bessard A, Frémin C, Ezan F, Fautrel A, Gailhouste L, Baffet G (2008). RNAi-mediated ERK2 knockdown inhibits growth of tumor cells in vitro and in vivo. *Oncogene*.

[100] Frémin C, Meloche S (2010). From basic research to clinical development of MEK1/2 inhibitors for cancer therapy. *Journal of Hematology and Oncology*.

[101] Kramer BW, Götz R, Rapp UR (2004). Use of mitogenic cascade blockers for treatment of C-Raf induced lung adenoma in vivo: CI-1040 strongly reduces growth and improves lung structure. *BMC Cancer*.

[102] Sebolt-Leopold JS, Dudley DT, Herrera R (1999). Blockade of the MAP kinase pathway suppresses growth of colon tumors in vivo. *Nature Medicine*.

[103] Collisson EA, De A, Suzuki H, Gambhir SS, Kolodney MS (2003). Treatment of metastatic melanoma with an orally available inhibitor of the Ras-Raf-MAPK cascade. *Cancer Research*.

[104] Brunet A, Pages G, Pouyssegur J (1994). Constitutively active mutants of MAP kinase kinase (MEK1) induce growth factor-relaxation and oncogenicity when expressed in fibroblasts. *Oncogene*.

[105] Cowley S, Paterson H, Kemp P, Marshall CJ (1994). Activation of MAP kinase kinase is necessary and sufficient for PC12 differentiation and for transformation of NIH 3T3 cells. *Cell*.

[106] Wellbrock C, Ogilvie L, Hedley D (2004). V599EB-RAF is an oncogene in melanocytes. *Cancer Research*.

[107] Hoshino R, Chatani Y, Yamori T (1999). Constitutive activation of the 41-/43-kDa mitogen-activated protein kinase signaling pathway in human tumors. *Oncogene*.

[108] Schmidt CM, McKillop IH, Cahill PA, Sitzmann JV (1997). Increased MAPK expression and activity in primary human hepatocellular carcinoma. *Biochemical and Biophysical Research Communications*.

[109] McKillop IH, Schmidt CM, Cahill PA, Sitzmann JV (1997). Altered expression of mitogen-activated protein kinases in a rat model of experimental hepatocellular carcinoma. *Hepatology*.

[110] Ito Y, Sasaki Y, Horimoto M (1998). Activation of mitogen-activated protein kinases/extracellular signal-regulated kinases in human hepatocellular carcinoma. *Hepatology*.

[111] Huynh H, Tuyen Nguyen TT, Pierce Chow KH, Tan PH, Soo KC, Tran E (2003). Over-expression of the mitogen-activated protein kinase (MAPK) kinase (MEK)-MAPK in hepatocellular carcinoma: its role in tumor progression and apoptosis. *BMC Gastroenterology*.

[112] Chen L, Shi Y, Jiang CY, Wei LX, Wang YL, Dai GH (2011). Expression and prognostic role of pan-Ras, Raf-1, pMEK1 and pERK1/2 in patients with hepatocellular carcinoma. *European Journal of Surgical Oncology*.

[113] Schmitz KJ, Wohlschlaeger J, Lang H (2008). Activation of the ERK and AKT signalling pathway predicts poor prognosis in hepatocellular carcinoma and ERK activation in cancer tissue is associated with hepatitis C virus infection. *Journal of Hepatology*.

[114] Malumbres M, Barbacid M (2003). RAS oncogenes: the first 30 years. *Nature Reviews Cancer*.

[115] Davies H, Bignell GR, Cox C (2002). Mutations of the BRAF gene in human cancer. *Nature*.

[116] Colombino M, Sperlongano P, Izzo F (2012). BRAF and PIK3CA genes are somatically mutated in hepatocellular carcinoma among patients from South Italy. *Cell Death & Disease*.

[117] Tannapfel A, Sommerer F, Benicke M (2003). Mutations of the BRAF gene in cholangiocarcinoma but not in hepatocellular carcinoma. *Gut*.

[118] Challen C, Guo K, Collier JD, Cavanagh D, Bassendine MF (1992). Infrequent point mutations in codons 12 and 61 of ras oncogenes in human hepatocellular carcinomas. *Journal of Hepatology*.

[119] Luo D, Liu QF, Gove C, Naomov NV, Su JJ, Williams R (1998). Analysis of N-ras gene mutation and p53 gene expression in human hepatocellular carcinomas. *World Journal of Gastroenterology*.

[120] Calvisi DF, Ladu S, Gorden A (2006). Ubiquitous activation of Ras and Jak/Stat pathways in human HCC. *Gastroenterology*.

[121] Fong CW, Chua MS, McKie AB (2006). Sprouty 2, an inhibitor of mitogen-activated protein kinase signaling, is down-regulated in hepatocellular carcinoma. *Cancer Research*.

[122] Yoshida T, Hisamoto T, Akiba J (2006). Spreds, inhibitors of the Ras/ERK signal transduction, are dysregulated in human hepatocellular carcinoma and linked to the malignant phenotype of tumors. *Oncogene*.

[123] Schuierer MM, Bataille F, Weiss TS, Hellerbrand C, Bosserhoff AK (2006). Raf kinase inhibitor protein is downregulated in hepatocellular carcinoma. *Oncology Reports*.

[124] Lee HC, Tian B, Sedivy JM, Wands JR, Kim M (2006). Loss of Raf kinase inhibitor protein promotes cell proliferation and migration of human hepatoma cells. *Gastroenterology*.

[125] Calvisi DF, Pinna F, Meloni F (2008). Dual-specificity phosphatase 1 ubiquitination in extracellular signal-regulated kinase-mediated control of growth in human hepatocellular carcinoma. *Cancer Research*.

[126] Hwang YH, Choi JY, Kim S (2004). Over-expression of c-raf-1 proto-oncogene in liver cirrhosis and hepatocellular carcinoma. *Hepatology Research*.

[127] Breuhahn K, Longerich T, Schirmacher P (2006). Dysregulation of growth factor signaling in human hepatocellular carcinoma. *Oncogene*.

[128] Tanaka H (1991). Immunohistochemical studies on epidermal growth factor receptor in hepatocellular carcinoma. *Nihon Shokakibyo Gakkai Zasshi*.

[129] Buckley AF, Burgart LJ, Sahai V, Kakar S (2008). Epidermal growth factor receptor expression and gene copy number in conventional hepatocellular carcinoma. *American Journal of Clinical Pathology*.

[130] Ito Y, Takeda T, Sakon M (2001). Expression and clinical significance of erb-B receptor family in hepatocellular carcinoma. *British Journal of Cancer*.

[131] Gangarosa LM, Sizemore N, Graves-Deal R, Oldham SM, Der CJ, Coffey RJ (1997). A Raf-independent epidermal growth factor receptor autocrine loop is necessary for Ras transformation of rat intestinal epithelial cells. *Journal of Biological Chemistry*.

[132] Schulze A, Lehmann K, Jefferies HBJ, McMahon M, Downward J (2001). Analysis of the transcriptional program induced by Raf in epithelial cells. *Genes and Development*.

[133] McCarthy SA, Samuels ML, Pritchard CA, Abraham JA, McMahon M (1995). Rapid induction of heparin-binding epidermal growth factor/diphtheria toxin receptor expression by Raf and Ras oncogenes. *Genes and Development*.

[134] Shimamura T, Saito S, Morita K (2000). Detection of vascular endothelial growth factor and its receptor expression in human hepatocellular carcinoma biopsy specimens. *Journal of Gastroenterology and Hepatology*.

[135] Poon RTP, Ho JWY, Tong CSW, Lau C, Ng IOL, Fan ST (2004). Prognostic significance of serum vascular endothelial growth factor and endostatin in patients with hepatocellular carcinoma. *British Journal of Surgery*.

[136] Semela D, Dufour JF (2004). Angiogenesis and hepatocellular carcinoma. *Journal of Hepatology*.

[137] Cariani E, Lasserre C, Seurin D (1988). Differential expression of insulin-like growth factor II mRNA in human primary liver cancers, benign liver tumors, and liver cirrhosis. *Cancer Research*.

[138] Breuhahn K, Vreden S, Haddad R (2004). Molecular profiling of human hepatocellular carcinoma defines mutually exclusive interferon regulation and insulin-like growth factor II overexpression. *Cancer Research*.

[139] Ueki T, Fujimoto J, Suzuki T, Yamamoto H, Okamoto E (1997). Expression of hepatocyte growth factor and its receptor c-met proto-oncogene in hepatocellular carcinoma. *Hepatology*.

[140] Erhardt A, Hassan M, Heintges T, Häussinger D (2002). Hepatitis C virus core protein induces cell proliferation and activates ERK, JNK, and p38 MAP kinases together with the MAP kinase phosphatase MKP-1 in a HepG2 Tet-Off cell line. *Virology*.

[141] Tsutsumi T, Suzuki T, Moriya K (2003). Hepatitis C virus core protein activates ERK and p38 MAPK in cooperation with ethanol in transgenic mice. *Hepatology*.

[142] Stöckl L, Berting A, Malkowski B, Foerste R, Hofschneider PH, Hildt E (2003). Integrity of c-Raf-1/MEK signal transduction cascade is essential for hepatitis B virus gene expression. *Oncogene*.

[143] Pang R, Lee TKW, Poon RTP (2007). Pin1 interacts with a specific serine-proline motif of hepatitis B virus X-protein to enhance hepatocarcinogenesis. *Gastroenterology*.

[144] Zhao LJ, Wang L, Ren H (2005). Hepatitis C virus E2 protein promotes human hepatoma cell proliferation through the MAPK/ERK signaling pathway via cellular receptors. *Experimental Cell Research*.

[145] Whittaker S, Marais R, Zhu AX (2010). The role of signaling pathways in the development and treatment of hepatocellular carcinoma. *Oncogene*.

[146] Menzo S, Clementi M, Alfani E (1993). Trans-activation of epidermal growth factor receptor gene by the hepatitis B virus X-gene product. *Virology*.

[147] Panteva M, Korkaya H, Jameel S (2003). Hepatitis viruses and the MAPK pathway: is this a survival strategy?. *Virus Research*.

[148] Hennig M, Yip-Schneider MT, Wentz S (2010). Targeting mitogen-activated protein Kinase Kinase with the inhibitor pd0325901 decreases hepatocellular carcinoma growth in vitro and in mouse model systems. *Hepatology*.

[149] Klein PJ, Schmidt CM, Wiesenauer CA (2006). The effects of a novel MEK inhibitor PD184161 on MEK-ERK signaling and growth in human liver cancer. *Neoplasia*.

[150] Huynh H, Ngo VC, Koong HN (2010). AZD6244 enhances the anti-tumor activity of sorafenib in ectopic and orthotopic models of human hepatocellular carcinoma (HCC). *Journal of Hepatology*.

[151] Gailhouste L, Ezan F, Bessard A (2010). RNAi-mediated MEK1 knock-down prevents ERK1/2 activation and abolishes human hepatocarcinoma growth in vitro and in vivo. *International Journal of Cancer*.

[152] Wiesenauer CA, Yip-Schneider MT, Wang Y, Schmidt CM (2004). Multiple anticancer effects of blocking MEK-ERK signaling in hepatocellular carcinoma. *Journal of the American College of Surgeons*.

[153] Bessard A, Frémin C, Ezan F, Coutant A, Baffet G (2007). MEK/ERK-dependent uPAR expression is required for motility via phosphorylation of P70S6K in human hepatocarcinoma cells. *Journal of Cellular Physiology*.

[154] Honma N, Genda T, Matsuda Y (2006). MEK/ERK signaling is a critical mediator for integrin-induced cell scattering in highly metastatic hepatocellular carcinoma cells. *Laboratory Investigation*.

[155] Guo K, Liu Y, Zhou H (2008). Involvement of protein kinase C *β*-extracellular signal-regulating kinase1/2/p38 mitogen-activated protein kinase-heat shock protein 27 activation in hepatocellular carcinoma cell motility and invasion. *Cancer Science*.

[156] Dai R, Chen R, Li H (2009). Cross-talk between PI3K/Akt and MEK/ERK pathways mediates endoplasmic reticulum stress-induced cell cycle progression and cell death in human hepatocellular carcinoma cells. *International Journal of Oncology*.

[157] Mitsui H, Takuwa N, Maruyama T (2001). The mek1-erk map kinase pathway and the pi 3-kinase-akt pathway independently mediate anti-apoptotic signals in hepg2 liver cancer cells. *International Journal of Cancer*.

[158] Lu Y, Cederbaum A (2007). The mode of cisplatin-induced cell death in CYP2E1-overexpressing HepG2 cells: modulation by ERK, ROS, glutathione, and thioredoxin. *Free Radical Biology and Medicine*.

[159] Caja L, Sancho P, Bertran E, Iglesias-Serret D, Gil J, Fabregat I (2009). Overactivation of the MEK/ERK pathway in liver tumor cells confers resistance to TGF-*β*-induced cell death through impairing up-regulation of the NADPH oxidase NOX4. *Cancer Research*.

[160] Coulouarn C, Factor VM, Thorgeirsson SS (2008). Transforming growth factor-*β* gene expression signature in mouse hepatocytes predicts clinical outcome in human cancer. *Hepatology*.

[161] Balmanno K, Cook SJ (2009). Tumour cell survival signalling by the ERK1/2 pathway. *Cell Death and Differentiation*.

[162] Liao M, Zhao J, Wang T, Duan J, Zhang Y, Deng X (2011). Role of bile salt in regulating Mcl-1 phosphorylation and chemoresistance in hepatocellular carcinoma cells. *Molecular Cancer*.

